# EPA and DHA inhibit LDL-induced upregulation of human adipose tissue NLRP3 inflammasome/IL-1β pathway and its association with diabetes risk factors

**DOI:** 10.1038/s41598-024-73672-6

**Published:** 2024-11-07

**Authors:** Valérie Lamantia, Simon Bissonnette, Myriam Beaudry, Yannick Cyr, Christine Des Rosiers, Alexis Baass, May Faraj

**Affiliations:** 1 Faculty of Medicine, Université de Montréal, Montréal, QC Canada; 2 Institut de Recherches Cliniques de Montréal (IRCM), 110, Avenue des Pins Ouest, Montréal, QC H2W 1R7 Canada; 3 Montréal Diabetes Research Center (MDRC), Montréal, QC Canada; 4 Montréal Heart Institute, Montréal, QC Canada; 5 Faculty of Medicine, McGill University, Montreal, QC Canada

**Keywords:** Marine-source omega-3 fatty acids, NLRP3 inflammasome and interleukin-1 beta, Plasma apoB, Human adipose tissue, Type 2 diabetes, Chronic inflammation, Type 2 diabetes, Translational research, Nutritional supplements

## Abstract

Elevated numbers of atherogenic lipoproteins (apoB) predict the incidence of type 2 diabetes (T2D). We reported that this may be mediated via the activation of the NLRP3 inflammasome, as low-density lipoproteins (LDL) induce interleukin-1 beta (IL-1β) secretion from human white adipose tissue (WAT) and macrophages. However, mitigating nutritional approaches remained unknown. We tested whether omega-3 eicosapentaenoic and docosahexaenoic acids (EPA and DHA) treat LDL-induced upregulation of WAT IL-1β-secretion and its relation to T2D risk factors*.* Twelve-week intervention with EPA and DHA (2.7 g/day, Webber Naturals) abolished baseline group-differences in WAT IL-1β-secretion between subjects with high-apoB (N = 17) and low-apoB (N = 16) separated around median plasma apoB. Post-intervention LDL failed to trigger IL-1β-secretion and inhibited it in lipopolysaccharide-stimulated WAT. Omega-3 supplementation also improved β-cell function and postprandial fat metabolism in association with higher blood EPA and mostly DHA. It also blunted the association of WAT *NLRP3* and *IL1B* expression and IL-1β-secretion with multiple cardiometabolic risk factors including adiposity. Ex vivo*,* EPA and DHA inhibited WAT IL-1β-secretion in a dose-dependent manner. In conclusion, EPA and DHA treat LDL-induced upregulation of WAT NLRP3 inflammasome/IL-1β pathway and related T2D risk factors. This may aid in the prevention of T2D and related morbidities in subjects with high-apoB.

*Clinical Trail Registration* ClinicalTrials.gov (NCT04496154): Omega-3 to Reduce Diabetes Risk in Subjects with High Number of Particles That Carry “Bad Cholesterol” in the Blood – Full Text View - ClinicalTrials.gov.

## Introduction

In 2021, 529 million people or 6.1% of the world’s population were living with diabetes, 96% of which were type 2 diabetes (T2D)^1^. T2D increases the risk for cardiovascular disease (CVD) and stroke by 2–4 fold^2^ and remains the leading cause of death and disability worldwide^1^. However, T2D is largely preventable^3^. Uncovering new mechanisms fueling T2D and their treatment are vital for the prevention of T2D and CVD and the extension of a healthy life span in humans.

The bidirectional communication between the immune and metabolic pathways is widely appreciated as a regulator of homeostasis as well as disease resulting from dysregulated inflammation^4,5^. The NLRP3 inflammasome is a key innate sensor of metabolic stress that is implicated in the pathology of CVD and more recently T2D (NLRP3 for Nucleotide-binding domain and Leucine-rich repeat Receptor, containing a Pyrin domain 3)^6–8^. Activation of the NLRP3 inflammasome leads to the secretion of interleukin-1 beta (IL-1β), which is reported to inhibit insulin signaling in various cell types including adipocytes and β-cells^6–8^.

For IL-1β to be secreted, two signals are needed. The first is a priming signal that occurs via the activation of the nuclear factor-κB pathway, downstream of membrane receptors such as toll-like receptors, scavenger receptors and cytokine receptors including IL-1 receptor^6–8^. This leads to the transcriptional upregulation of *NLRP3* and *IL1B* or post-translational modification of NLRP3 independent of its transcription^9,10^. Priming signals in macrophages include microbial lipopolysaccharide (LPS)^7,11^, palmitate^12^, and oxidized LDL^13^. The second signal is an activation signal that promotes the assembly of the inflammasome subunits, activation of caspase-1, cleavage of pro-IL-1β, and secretion of IL-1β. Activation signals in LPS-primed β-cells and macrophages include islet amyloid polypeptide oligomers^8^, ceramide^14^, adenosine triphosphate (ATP)^15^ and palmitate^11^.

While the upregulation of the NLRP3 inflammasome in white adipose tissue (WAT) is believed to promote metabolic stress and T2D, endogenous signals that stimulate the NLRP3 inflammasome in human WAT triggering IL-1β-secretion were unclear. Recently, we reported that native low-density lipoproteins (LDL), the most common form of atherogenic lipoproteins, are priming signals of the NLRP3 inflammasome that lead to IL-1β-secretion from human WAT and monocyte-derived macrophages^16^. Subjects with high plasma numbers of apoB-lipoproteins (high-apoB) had higher WAT IL-1β-secretion than subjects with low-apoB^16^. Importantly, WAT IL-1β-secretion induced by LDL (without/with ATP) was associated with risk factors for T2D mostly in subjects with high-apoB not low-apoB^16^. However, nutritional approaches that mitigate LDL-induced upregulation of human WAT NLRP3 inflammasome/ IL-1β pathway remained to be established.

Marine-source omega-3 eicosapentaenoic acid (EPA) and docosahexaenoic acid (DHA) were reported to inhibit the priming and activation of the NLRP3 inflammasome in macrophages *in vitro*^17,18^. Moreover, a meta-analysis in healthy adults reported that supplementation with 1.2–5.2 g/day of EPA and DHA for 6–24 weeks (EPA:DHA ratio of ~ 1.5:1 to 2.4:1) reduces IL-1β-secretion from cultured LPS-primed human monocytes^19^. Thus, we tested the hypotheses that EPA and DHA treat LDL-induced upregulation of the WAT NLRP3 inflammasome/ IL-1β pathway and its relation to T2D risk factors in humans.

## Methodology

### Study objectives, population and design

This work represents the post-intervention outcomes of a clinical trial with 12-week supplementation with EPA and DHA that was conducted at the Montréal Clinical Research Institute (IRCM). The central hypothesis of the trial was that apoB-lipoproteins act as metabolic danger-associated molecular patterns that activate the NLRP3 inflammasome in WAT leading to WAT dysfunction and associated risks for T2D in humans, which can be treated by EPA and DHA. Subject recruitment was completed between 2013 and 2019. Nine-hundred and thirty subjects were screened, of whom 40 subjects were included (N = 27 females and N = 13 males)^16^. Baseline data reporting the effects of LDL on the WAT NLRP3 inflammasome/ IL-1β pathway in vivo and ex vivo were recently published^16^.

The hypotheses tested post-intervention were that supplementation with EPA and DHA 1) induces a greater reduction in WAT IL-1β-secretion in subjects with high-apoB than low-apoB eliminating baseline group-differences (primary hypothesis), 2) induces a greater reduction in risk factors for T2D in subjects with high-apoB than low-apoB (secondary hypothesis), and 3) reduces the baseline associations of WAT IL-1β-secretion with risk factors for T2D (secondary hypothesis). Moreover, we tested whether EPA and DHA inhibit LDL-induced priming and/or activation of WAT NLRP3 inflammasome ex vivo (secondary hypothesis*)*. The sample size of N = 20/group was powered to test baseline and post-intervention group-differences in the primary outcome (WAT IL-1β-secretion)^16^.

Inclusion and exclusion criteria were reported^16^. Briefly, the trial included 45–74-year-old non-smoker males and postmenopausal females with body mass index greater than 20 kg/m^2^, sedentary lifestyle and low-moderate alcohol consumption. Exclusion criteria were chronic disease (e.g. CVD, diabetes, inflammatory), medications affecting metabolism, and allergy to seafood or fish. The trial was registered at ClinicalTrial.org (identifier: NCT04496154) on 03/08/2020. Sample analysis was blinded using subjects’ identification number.

Subjects were placed on a 4-week weight-stabilization period after which baseline outcome measures were conducted^16^. Three-day food reports were completed by the subjects and verified by the study dietitian. Assessment of the risk factors for T2D were conducted on 2 separate days, 1–4 weeks apart. Baseline measures were repeated following the intervention and post-intervention food records were completed in the last week of the intervention.

### Intervention with EPA and DHA

Subjects followed a 12-week supplementation with 2.7 g/day EPA and DHA (3 softgels of Webbers Naturals Triple Strength Omega-3). Each softgel contains 1425 mg fish oil concentrate from anchovy, sardines and/or mackerel providing 600 mg EPA and 300 mg DHA in ethyl ester form. This supplementation received the Internationally Verified Omega-3 certification. This certification program ensures that the marine oil meets 90–165% of the label claim for EPA and DHA, has an oxidation limit (Totox value) ≤ 25 mEq/kg, and ≤ accepted limits for environmental toxins, heavy metals and microbiological contamination^20^. Subjects received their supply of omega-3 and had their body weight recorded monthly at IRCM. They were advised to consume the softgels with food and to preserve the opened bottle in the fridge to reduce light exposure and oxidation. They were also instructed to maintain their habitual dietary and physical activity during the intervention.

### First testing day for body composition, glucose-induced insulin secretion (GIIS) and insulin sensitivity (IS)

Body composition was measured by dual energy X-ray absorptiometry (GE Healthcare, Little Chalfont, UK), after which GIIS and IS were measured by gold-standard Botnia clamps as standard^16,21–28^. Briefly, GIIS and C-peptide secretion were measured during a 1-h intravenous glucose tolerance test (IVGTT). The first- and second-phase insulin and C-peptide secretions were calculated as the area under the curve (AUC) of their plasma levels during the first 10 min and the following 50 min of the IVGTT, respectively. The IVGTT was immediately followed by a 3-h hyperinsulinemic euglycemic clamp, during which IS was measured as the glucose infusion rate divided by steady state plasma insulin (M/I_clamp_). The disposition index (DI) was calculated as the 1st phase or total C-peptide secretion_IVGTT_ multiplied by IS (M/I_clamp_). Fasting blood samples collected from this day were used to isolate native LDL and measure circulating fatty acid (FA) profile.

### Second testing day for basal metabolic rate and postprandial plasma fat clearance and for the collection of WAT biopsies

Basal metabolic rate was measured after 12-h fast by indirect calorimetry (Vmax Encore; Carefusion). Subjects then consumed a standardized high-fat meal (600 kcal/m^2^, 68% fat, 36% saturated fat, 18% carbohydrate)^16,21,23–26,28^. Postprandial plasma clearance rates of fat and chylomicrons were calculated as the AUC of plasma triglycerides (TG) and apoB48 over 6 h, *respectively*. Fasting WAT biopsies were collected from the right hip by needle-liposuction under local anesthesia (Xylocaine 20 mg/mL, AstraZeneca) using a syringe. Biopsies were conducted between 7:30 and 9:30 am over a period of 2–2.5 min per biopsy. WAT biopsies were immediately washed with antibiotic/antifungal-supplemented Hanks’ Balanced Salt solution at 37 °C and processed as follows when sufficient yield was available ^16,21,23–26,28^: a portion was snap-frozen in liquid nitrogen for the measurement of mRNA and protein expressions, and another was used fresh within 45–60 min after the WAT biopsy to assess IL-1β-secretion ex vivo.

### Plasma and WAT-secreted parameters

Plasma lipids, apoB and apoA1 were measured by an automated analyzer (Cobas Integra 400; Roche Diagnostics), plasma apoB48 and proprotein convertase subtilisin/kexin type 9 (PCSK9) by enzyme-linked immunosorbent assay kits (BioVendor and CircuLex MBL International, *respectively*), plasma glucose by an automated analyzer (YSI 2300 STAT Plus) and serum insulin and C-peptide by radioimmunoassay kits (Millipore Corporation). IL-1β accumulation in WAT culture medium was measured by alpha-LISA® kits (Perkin Elmer, Canada)^16^.

### Plasma and red blood cell (RBC) FA profile

To assess the compliance to the intervention and to have an objective measure of EPA and DHA bioavailability, the concentrations of FA in the phospholipid (PL) layer of plasma (i.e. lipoproteins) and RBC (i.e. long-term bioavailability) were measured by gas chromatography–mass spectrometry as published^16,27^. Briefly, PL were separated on an aminopropyl column, and the FA were converted to methyl esters for analysis. The FA concentrations were calculated using external and internal isotope-labeled standards and expressed in molar concentrations and percent (%) of total FA. The post-intervention % shift in omega-3 FA in RBC was calculated by subtracting the baseline from the post-intervention % omega-3 FA in RBC.

### Regulation of WAT NLRP3 inflammasome/IL-1β pathway

WAT samples were incubated for 4 h with the following inflammasome priming conditions^16^: medium alone or supplemented with native LDL or LPS. Medium was removed and WAT samples were washed and re-incubated for 3 h with the following activating conditions: medium alone or supplemented with native LDL or ATP. Medium accumulation of IL-1β was then quantified (termed IL-1β-secretion). The medium used was Dulbecco’s-Modified Eagle Medium containing 5% fetal bovine serum (Gibco/Thermo Fisher). Native LDL was isolated from fasting blood and used within 1–4 weeks on subject’s own WAT^16^. LDL of 1.2 g/L apoB was used as it corresponds to the 75^th^ percentile in Canadians^29^, was previously used to induce human WAT dysfunction^23,28^, and represents average levels of our cohorts with high-apoB^24,28,30,31^. LPS was used as the positive control for the inflammasome priming (Sigma-Aldrich L4591, 0.3 μg/ml) and ATP as that for activation (Sigma-Aldrich A2383, 3 mmol/L) as determined in baseline pilot kinetic studies^16^. WAT experiments used 5–10 mg WAT/well and 2–4 wells/condition^16^.

To assess the direct effect of EPA and DHA on WAT IL-1β-secretion ex vivo, EPA and DHA were purchased from Sigma Aldrich and used at a ratio of 2:1 as in the omega-3 softgels. They were co-incubated with LDL, LPS and/or ATP during the priming and activation periods. Their effects were compared to equal concentrations of palmitate and oleate. All FA were bound to albumin (0.105 mmol/L, US biological, low endotoxin), sterilized, sealed under nitrogen and stored at − 80 °C. Final concentrations of FA (50, 100, and 200 µmol/L) were measured by a commercial kit (Fujifilm Wako Pure Chemical Corporation)^32^.

### WAT mRNA and protein expressions

mRNA expressions of genes related to inflammation and the NLRP3 inflammasome subunits (*IL1B*, *NLRP3*, *CASP1,ADGRE1*, *MCP1*, *IL10)* and WAT lipid metabolism and function* (LDLR, CD36, HMGCR, SREBP1c, SREBP2, PPARG, ADIPOQ)* were analyzed by real-time polymerase chain reaction using RotorGene Q (Qiagen) using *HPRT* as a reference gene^16^. WAT proteins were extracted in radioimmunoprecipitation assay buffer and pro-IL-1β was quantified by western blot using an internal control made with pooled WAT from 5 subjects. The list of primers and antibodies were previously reported^25,26^.

### Statistical analysis

**Fig. 1:**
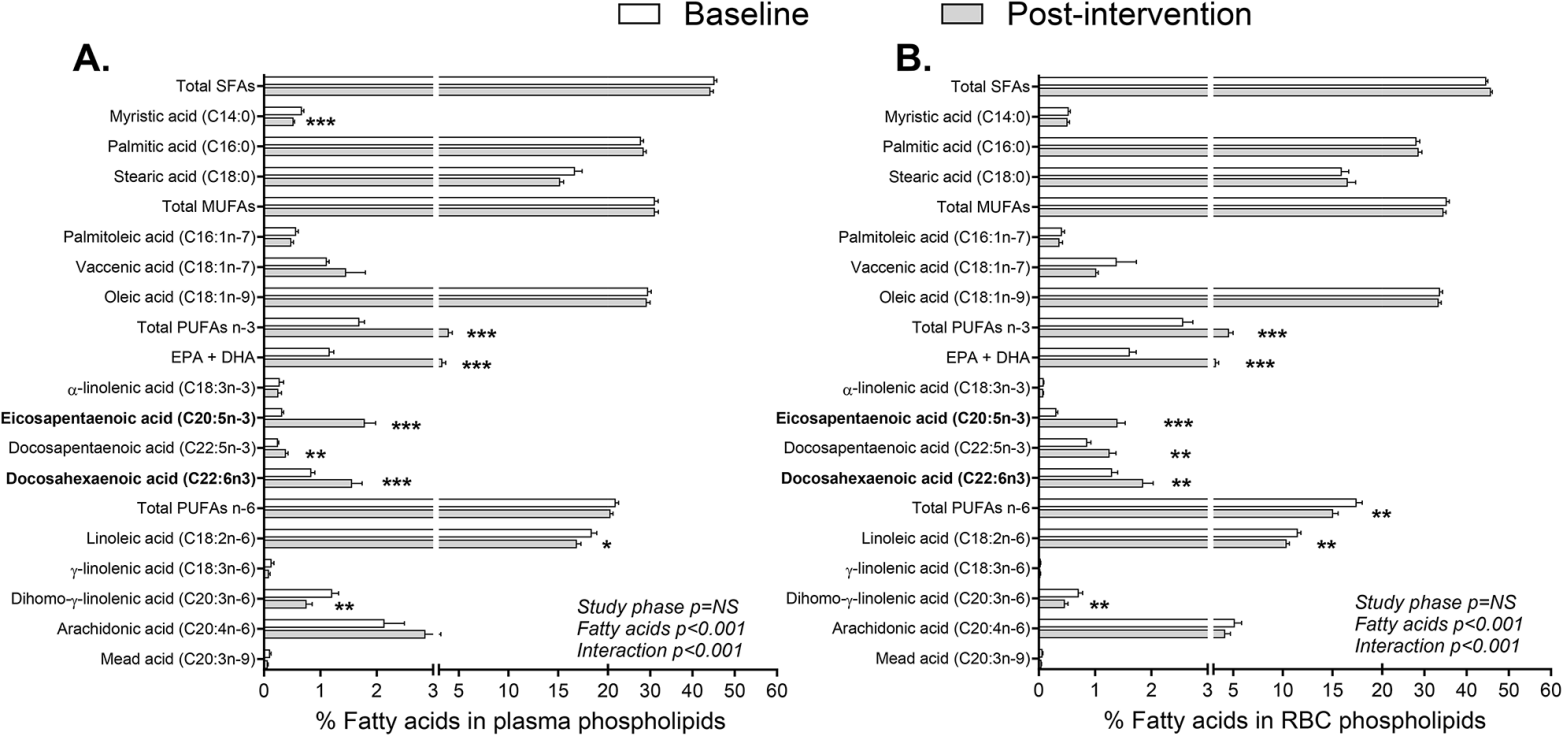
Percent FA in plasma and RBC PL at baseline and post-intervention: Baseline and post-intervention % FA in plasma PL (**A**) and RBC PL (**B**) in all subjects (N = 33) who completed the 12-week supplementation with 2.7 g/d EPA and DHA. N.B. Mead acid was excluded from analysis as only N = 2 had measurable post-intervention data in RBC. * for *p* < 0.05, ** for *p* < 0.01 and *** for *p* < 0.001 versus baseline.

**Fig. 2:**
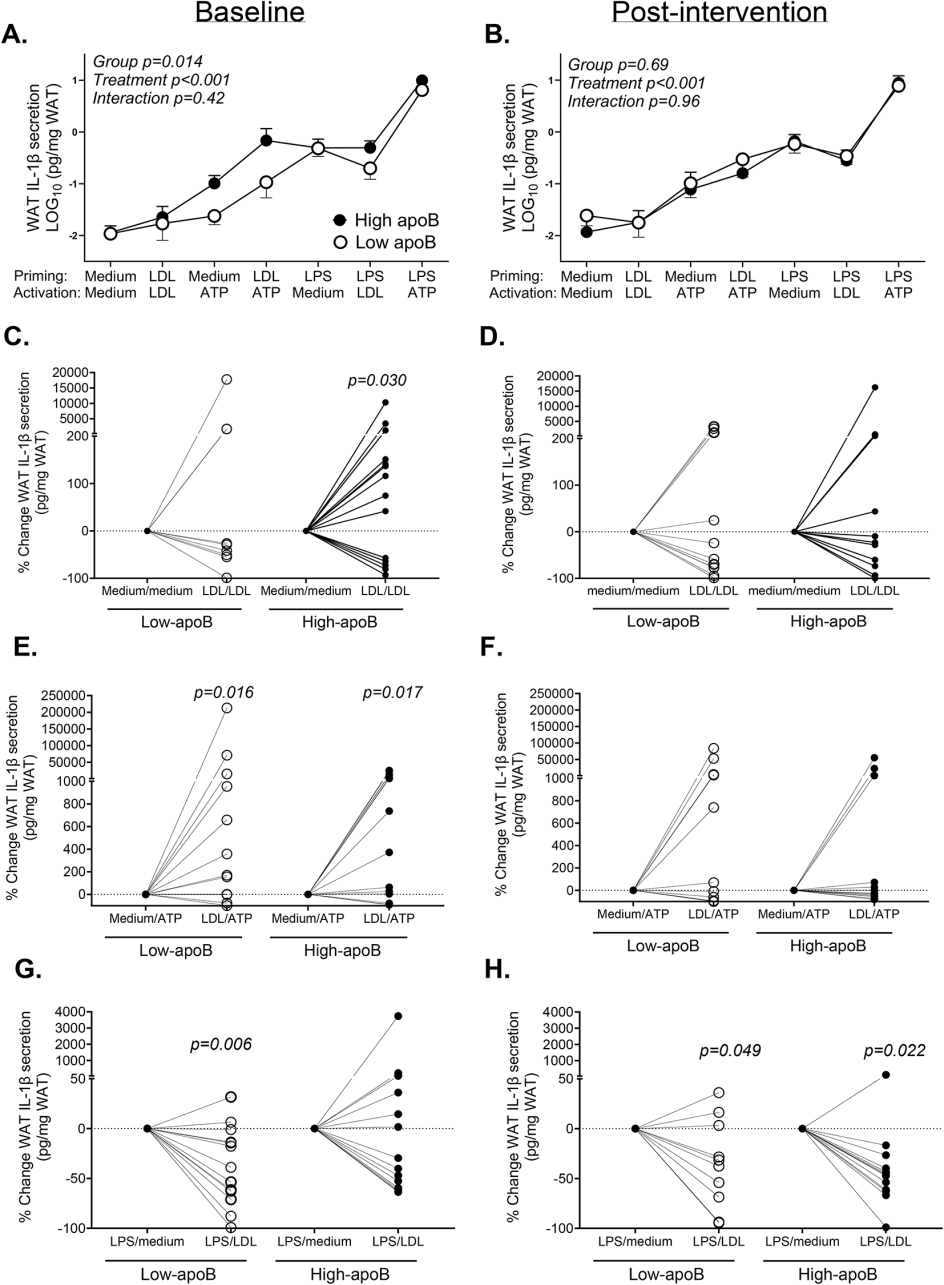
WAT IL-1β-secretion at baseline and post-intervention: WAT IL-1β-secretion induced by the 7-incubation conditions with LDL, LPS and/or ATP at baseline (**A**) and post-intervention (**B**), and % change in WAT IL-1β-secretion induced by LDL/LDL versus medium/medium (negative control) at baseline (**C**) and post-intervention (**D**), by LDL/ATP versus medium/ATP at baseline (**E**) and post-intervention (**F**), and by LPS/LDL versus LPS/medium at baseline (**G**) and post-intervention (**H**) in subjects with low-apoB (N = 13) and high-apoB (baseline N = 15, post-intervention N = 13) who completed the intervention.

**Fig. 3:**
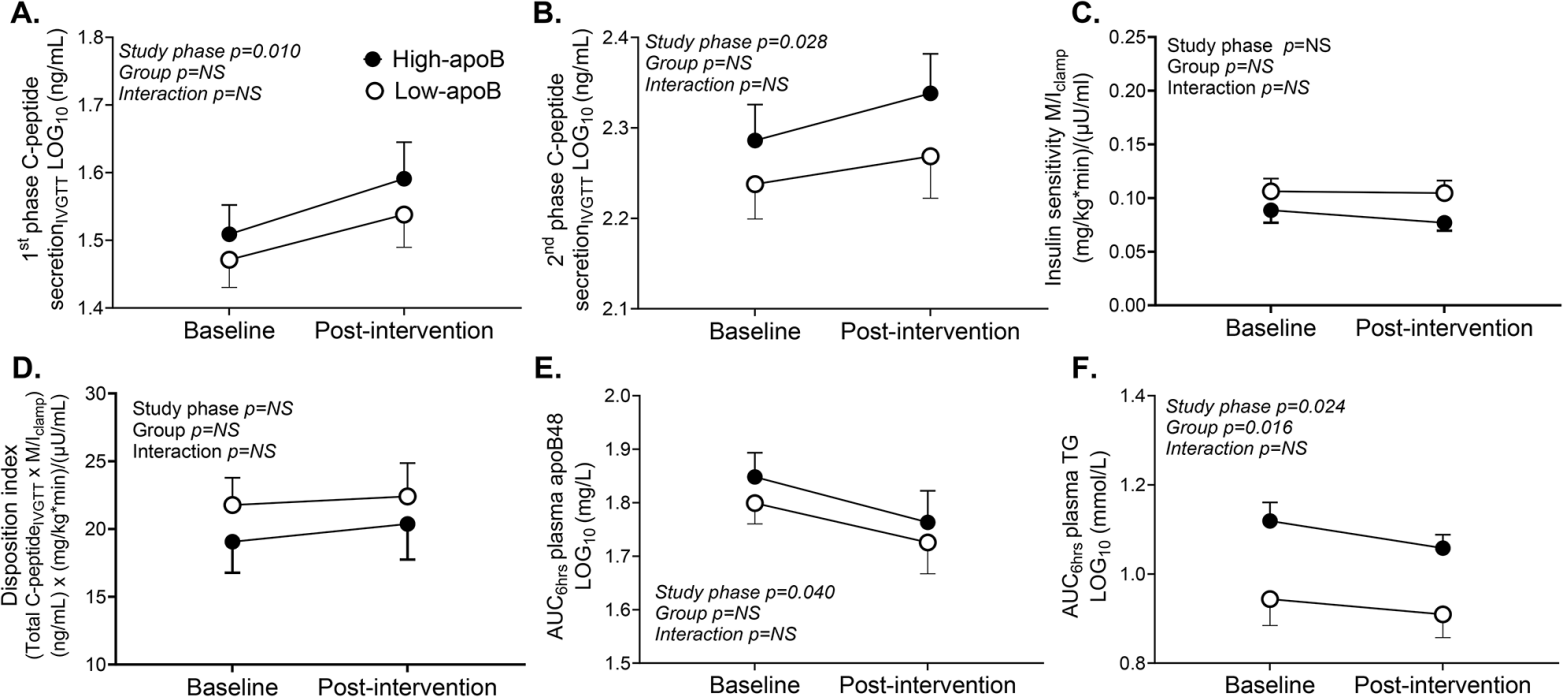
Risk factors for T2D at baseline and post-intervention: Group-differences at baseline and post-intervention in 1st phase C-peptide secretion (**A**), 2nd phase C-peptide secretion (**B**), insulin sensitivity (**C**), total disposition index (**D**), AUC_6hrs_ postprandial plasma apoB48 (**E**), and AUC_6hrs_ postprandial plasma TG (**F**) in subjects with low-apoB (N = 16) and high-apoB (N = 17) who completed the intervention, except for panels **E** and **F** where N = 15 for low-apoB and N = 16 for high-apoB for missing data.

**Fig. 4:**
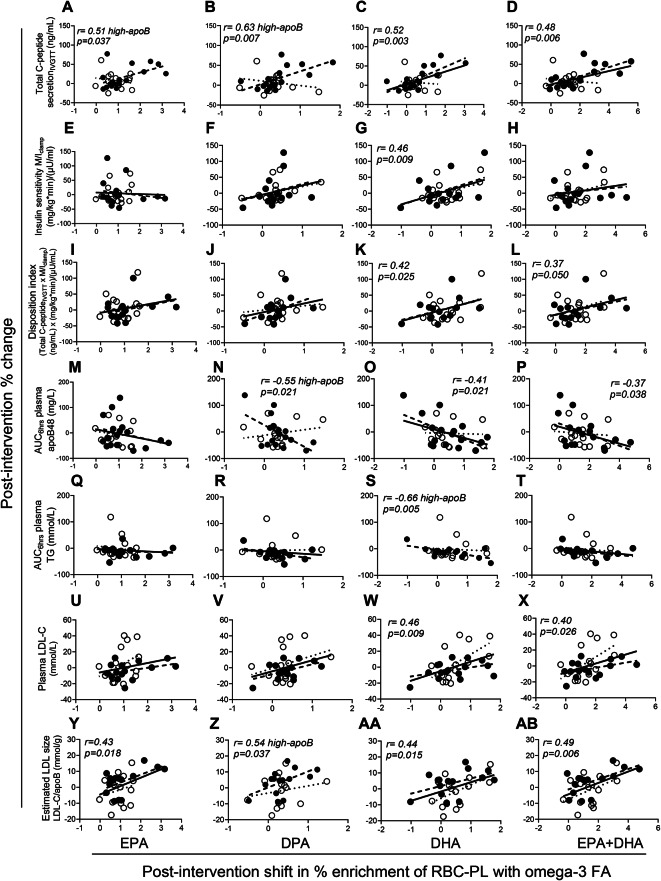
Association of post-intervention % shift in RBC PL omega-3 FA with % change in T2D risk factors: Pearson correlation of % shift in EPA, DPA, DHA or EPA + DHA with % change in total C-peptide secretion (**A–D**), insulin sensitivity (**E–H**), total disposition index (**I–L**), AUC_6hrs_ postprandial plasma apoB48 (**M–P**), AUC_6hrs_ postprandial plasma TG (**Q–T**), fasting plasma LDL-C (**U-X**) and estimated LDL size (**Y-AB**) in subjects with low-apoB (N = 16, open circles, dotted regression lines) and high-apoB (N = 17, closed circles, dashed regression lines) who completed the 12-week supplementation with EPA and DHA, except for panels **M-AB** where N = 15 for low-apoB and N = 16 for high-apoB for missing data. Solid regression line represents pooled data for all subjects.

**Fig. 5:**
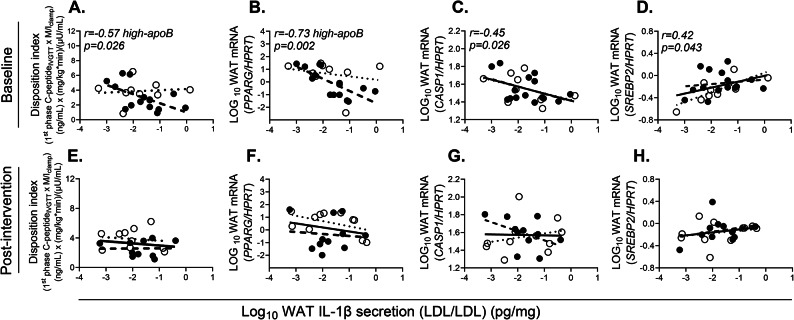
Association of LDL/LDL-induced WAT IL-1β-secretion with T2D risk factors at baseline and post-intervention: Pearson correlation of LDL/LDL-induced WAT IL-1β-secretion with 1st phase disposition index (**A**), WAT mRNA expression of *PPARG* (**B**), *CASP1* (**C**) and *SREBP2* (**D**) normalized for *HPRT* at baseline in subjects with low-apoB (N = 9, open circles, dotted regression lines) and high-apoB (N = 15, closed circles, dashed regression lines) who completed the intervention, and with the same parameters post-intervention (**E–H**) in subjects with low-apoB (N = 12) and high-apoB (N = 13). Solid regression line represents pooled data for all subjects.

**Fig. 6:**
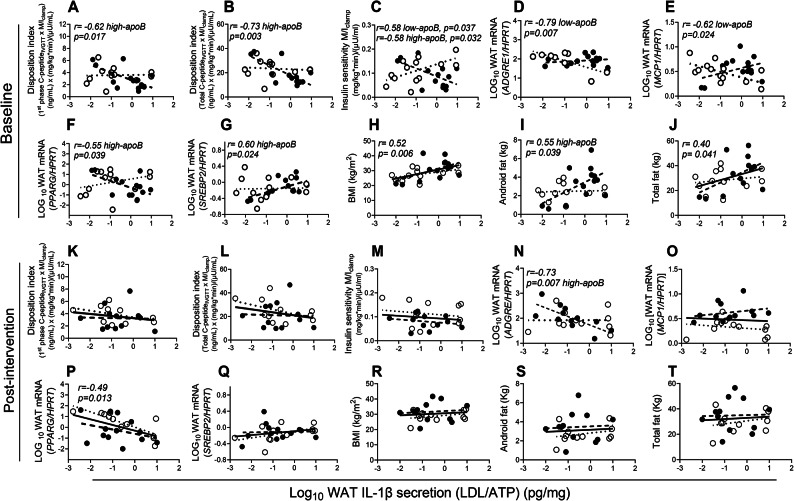
Association of LDL/ATP-induced WAT IL-1β-secretion with T2D risk factors at baseline and post-intervention: Pearson correlation of baseline LDL/ATP-induced WAT IL-1β-secretion with 1st phase disposition index (**A**), total disposition index (**B**),  insulin sensitivity (**C**), WAT mRNA expression of *ADGRE1* (**D**), *MCP1* (**E**), *PPARG* (**F**), and *SREBP2* (**G**) normalized for *HPRT*, BMI (**H**), android fat (**I**), and total body fat (**J**) at baseline in subjects with low-apoB (N = 13, open circles, dotted regression lines) and high-apoB (N = 14, closed circles, dashed regression lines) who completed the intervention, and with the same parameters post-intervention (**K–T**) in subjects with low-apoB (N = 12) and high-apoB (N = 13). Solid regression line represents pooled data for all subjects.

**Fig. 7:**
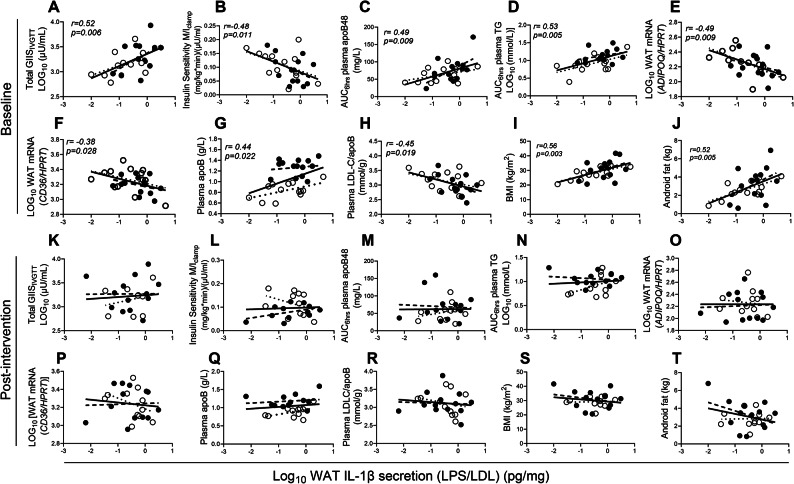
Association of LPS/LDL-induced WAT IL-1β-secretion with T2D risk factors at baseline and post-intervention: Pearson correlation of baseline LPS/LDL-induced WAT IL-1β-secretion with total GIIS_IVGTT_ (**A**),  insulin sensitivity (**B**), AUC_6hrs_ postprandial plasma apoB48 (**C**), AUC_6hrs_ postprandial plasma TG (**D**), fasting WAT mRNA expression of *ADIPOQ* (**E**) and *CD36* (**F**) normalized for *HPRT,* fasting plasma apoB (**G**), estimated LDL size (**H**) BMI (**I**), and android fat (**J**) in subjects with low-apoB (N = 13, open circles, dotted regression lines) and high-apoB (N = 14, closed circles, dashed regression lines) who completed the intervention, and with the same parameters post-intervention (**K–T**) in subjects with low-apoB (N = 11) and high-apoB (N = 13). Solid regression line represents pooled data for all subjects.

**Fig. 8:**
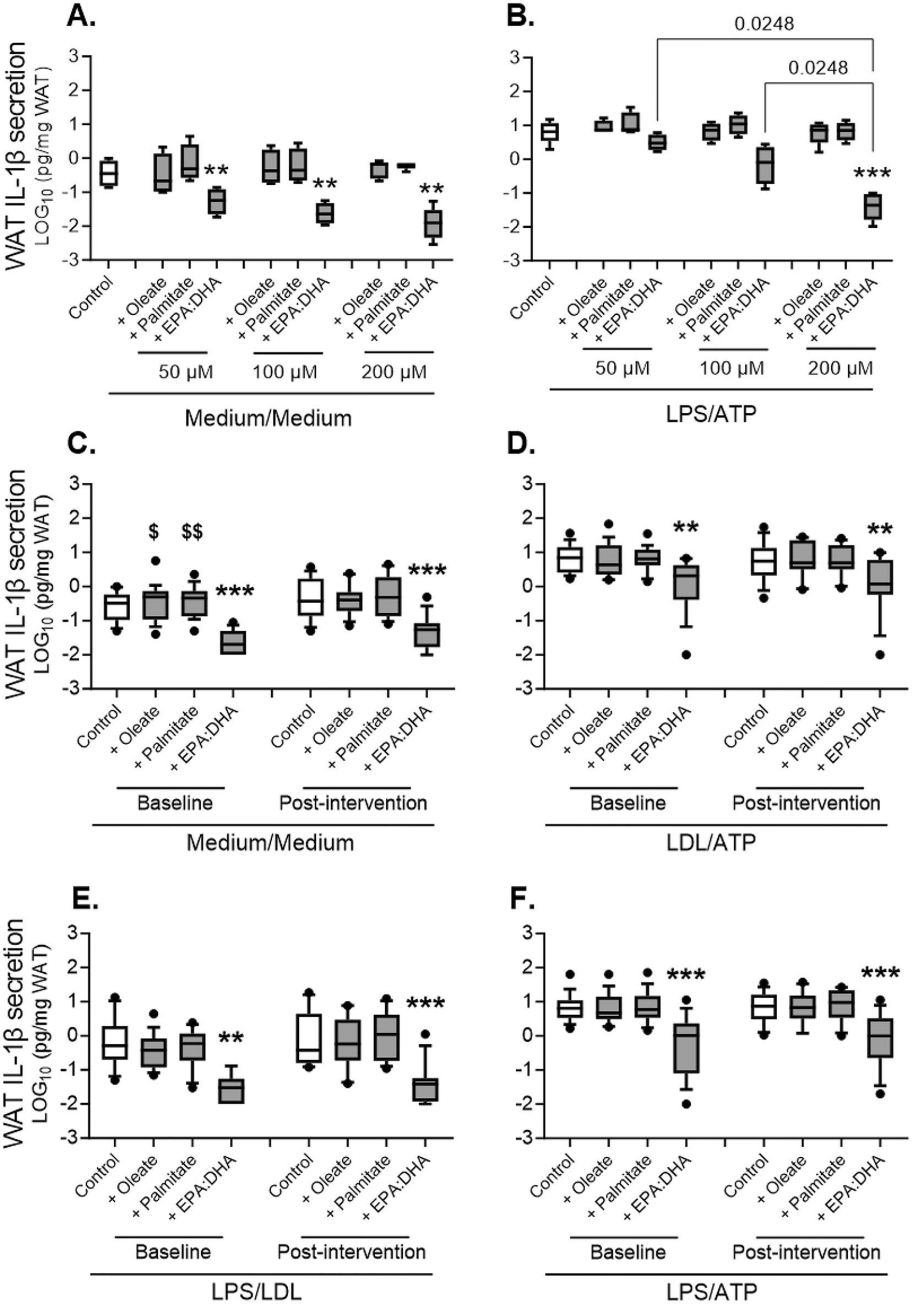
Effects of EPA and DHA on WAT IL-1β-secretion ex vivo: Effects of 50, 100 or 200 µmol/L EPA:DHA (2:1) versus equal concentrations of oleate and palmitate on IL-1β-secretion in unstimulated WAT (**A**) and LPS/ATP-stimulated WAT (**B**), and effects of 200 µmol/L EPA:DHA (2:1) versus oleate and palmitate on IL-1β-secretion at baseline and post-intervention in unstimulated WAT (**C**), LDL/ATP-stimulated WAT (**D**), LPS/LDL-stimulated WAT (**E**) and LPS/ATP-stimulated WAT (**F**) in subjects who completed the intervention. Panels **A, B** included 5 subjects at baseline (N = 1 low-apoB and N = 4 high-apoB) and panels **C–F** included 18 subjects at baseline (N = 9 low-apoB and N = 9 high-apoB) and 14 subjects post-intervention (N = 6 low-apoB and N = 8 high-apoB). * for *p* < 0.05, ** for *p* < 0.01 and *** for *p* < 0.001 versus control (0.105 µmol/L albumin in 5% fetal bovine serum medium) or similar concentrations of oleate or palmitate, and ^$^ for *p* < 0.05, ^$$^ for *p* < 0.01 versus control.

Data are presented as mean ± standard error of the mean. As WAT and/or LDL were insufficient to complete all experiments in some subjects, group-differences between subjects with low-apoB and high-apoB were analyzed by 2-way ANOVA for repeated measures based on a mixed-model with interaction (Figs.1, 2A–B, and 3). When the interaction was significant, inter or intra-subject differences were further analyzed by unpaired or paired t-test, *respectively.* The effects of FA on WAT IL-1β-secretion ex vivo were analyzed by 1-way ANOVA for repeated-measures based on a mixed-model (Fig. 8). ANOVA analyses were conducted with Geisser-Greenhouse correction and with controlling for false-discovery rate. Data with large intersubject-variability (i.e. raw data for IL-1β-secretion, gene expression, insulin and C-peptide secretions_IVGTT_, and AUC_6hrs_ of plasma TG) were LOG_10_ transformed before being used in analyses. Non-parametric Wilcoxon signed rank test was used when data could not be LOG_10_ transformed (i.e. % changes in Fig. 2C–H). Pearson correlation was used to examine the association between variables in the low-apoB and high-apoB groups separately and data were pooled when no group-differences in the regression lines existed (Figs. 4, 5, 6, and 7). Statistical analyses were performed using SPSS (V26) and GraphPad Prism (V 9.4) with significance set at *p* < 0.05.

## Results

Forty subjects were included in this trial. Three subjects dropped out for lack of time/personal reasons, 2 for being unreachable, 1 for being diagnosed with cancer, and 1 for inability to swallow the omega-3 softgels. One female did not undergo the second testing day with WAT biopsies for lack of time and another was excluded by the investigators for oversensitivity to the insulin infusion during the clamp (Supplementary Fig. S1). Thus, this analysis was conducted on 33 subjects who completed the trial and were stratified based on baseline median fasting plasma apoB per sex.

Supplementation with EPA and DHA increased % EPA, docosapentaenoic acid (DPA, intermediate between EPA and DHA in the biosynthesis pathway^33^), DHA and total omega-3 FA in plasma and RBC confirming subject compliance (Fig. 1, Table 1). There was also a post-intervention decrease in % omega-6 FA and omega-6/omega-3 ratio. Similar changes in omega-3 and omega-6 FA were observed using their molar concentrations (Supplementary Fig. S2). Subjects with high-apoB had higher % EPA and DHA and lower omega-6/omega-3 ratio in RBC (Table 1). Plasma % EPA and/or DHA, % total omega-3, % total omega-6, and omega-6/omega-3 ratio were positively correlated with their counterparts in RBC (Supplementary Fig S3). There were no post-intervention changes in body composition or fasting metabolic parameters, including apoB, except for a decrease in systolic blood pressure and plasma FA and PCSK9 and an increase in plasma high-density lipoprotein cholesterol (HDL-C) and apoB/PCSK9 ratio (Table 1). Subjects with high-apoB had higher fasting plasma total cholesterol, LDL-C, TG and apoB/PCSK9 ratio and % fat intake and lower % carbohydrate intake. There were no post-intervention changes in dietary intake and expenditure in either group.

**Table 1:** Baseline and post-intervention data of subjects with low-apoB and high-apoB.

	Low-apoB (N = 16)			High-apoB (N = 17)			*P* value		
	Baseline	Post-intervention	Change	Baseline	Post-intervention	Change	Time effect	Group effect	Interaction
Plasma apoB (g/L)^1^	0.85 ± 0.03	0.88 ± 0.04	0.04 ± 0.04	1.23 ± 0.04*	1.18 ± 0.04*	− 0.06 ± 0.03	0.723	**< 0.001**	**0.049**
*Anthropometric parameters*									
Age (yrs)	57.8 ± 1.8	58.3 ± 1.7	0.50 ± 0.13	59.5 ± 2.2	59.8 ± 2.1	0.29 ± 0.11	**< 0.001**	0.579	0.239
Weight (kg)	77.2 ± 2.8	77.3 ± 2.7	0.15 ± 0.55	84.7 ± 5.4	84.9 ± 5.4	0.13 ± 0.42	0.686	0.230	0.977
BMI (kg/m^2^)	28.3 ± 0.9	28.3 ± 0.9	0.02 ± 0.21	31.2 ± 1.6	31.2 ± 1.6	0.02 ± 0.15	0.903	0.144	0.997
Fat mass (kg)	28.8 ± 2.1	28.9 ± 2.2	0.15 ± 0.43	33.6 ± 3.2	33.5 ± 3.2	− 0.12 ± 0.38	0.963	0.244	0.636
Lean body mass (kg)	45.4 ± 2.4	45.5 ± 2.4	0.05 ± 0.26	48.1 ± 2.9	48.5 ± 3.0	0.34 ± 0.20	0.232	0.462	0.372
Android fat mass (kg)	2.57 ± 0.22	2.59 ± 0.24	0.02 ± 0.07	3.28 ± 0.40	3.21 ± 0.38	− 0.06 ± 0.06	0.623	0.157	0.393
Gynoid fat mass (kg)	4.84 ± 0.35	4.97 ± 0.40	0.13 ± 0.08	5.38 ± 0.53	5.32 ± 0.49	− 0.06 ± 0.08	0.580	0.486	0.106
Android/gynoid	0.54 ± 0.04	0.53 ± 0.03	− 0.01 ± 0.01	0.58 ± 0.04	0.58 ± 0.04	− 0.01 ± 0.01	0.365	0.356	0.798
Basal metabolic rate (kcal/d)^1^	1376 ± 48	1334 ± 79	− 50.3 ± 72.0	1500 ± 89	1560 ± 106	39.8 ± 47.3	0.903	0.120	0.298
Energy intake (kcal)^2^	2143 ± 147	2066 ± 179	− 77 ± 154	2355 ± 141	2182 ± 139	− 207 ± 77	0.111	0.457	0.373
Fat intake (% kcal)^2^	32.5 ± 1.4	34.4 ± 2.1	1.87 ± 2.39	38.1 ± 1.5	37.4 ± 1.6	− 0.82 ± 1.26	0.699	**0.031**	0.328
Carbohydrate intake (% kcal)^2^	51.2 ± 1.9	49.1 ± 2.5	− 2.08 ± 2.89	44.2 ± 1.4	46.0 ± 1.8	1.74 ± 1.74	0.920	**0.028**	0.266
Protein intake (% kcal)^2^	16.3 ± 0.6	17.6 ± 0.7	1.25 ± 0.84	16.2 ± 1.0	16.2 ± 0.6	0.11 ± 0.98	0.301	0.336	0.383
Alcohol (% kcal)^2^	1.75 ± 0.50	0.93 ± 0.45	− 0.82 ± 0.47	2.74 ± 0.97	1.43 ± 0.63	− 1.39 ± 1.00	0.055	0.342	0.611
*Fasting parameters*									
SBP (mm Hg)^3^	118 ± 2	114 ± 3	− 2.21 ± 3.72	126 ± 3	120 ± 3	− 6.18 ± 2.07	**0.048**	0.130	0.338
DBP (mm Hg)^3^	73.8 ± 2.2	72.4 ± 1.9	0.29 ± 2.47	76.2 ± 1.9	71.5 ± 2.1	− 4.76 ± 1.43	0.113	0.976	0.075
Plasma glucose (mmol/L)	5.13 ± 0.19	5.02 ± 0.14	− 0.11 ± 0.16	5.06 ± 0.10	5.22 ± 0.12	0.17 ± 0.08	0.741	0.699	0.125
Plasma insulin (μU/mL)^4^	11.6 ± 2.3	9.8 ± 1.7	− 2.40 ± 0.83	11.7 ± 1.9	13.0 ± 1.9	1.29 ± 1.95	0.676	0.591	0.126
HOMA-IR (mmol/L)x(μU/mL)	2.83 ± 0.73	2.09 ± 0.38	− 0.74 ± 0.42	2.66 ± 0.49	3.06 ± 0.46	0.40 ± 0.52	0.608	0.557	0.098
Plasma cholesterol (mmol/L)^1^	4.66 ± 0.15	4.85 ± 0.13	0.21 ± 0.14	5.83 ± 0.17*	5.64 ± 0.18*	− 0.19 ± 0.13	0.903	**< 0.001**	**0.045**
Plasma LDL-C (mmol/L)^1^	2.65 ± 0.12	2.73 ± 0.14	0.08 ± 0.14	3.74 ± 0.14	3.61 ± 0.14	− 0.15 ± 0.11	0.701	**< 0.001**	0.185
Plasma LDL-C/apoB (mmol/g)^1^	3.15 ± 0.08	3.09 ± 0.08	− 0.07 ± 0.08	3.05 ± 0.08	3.07 ± 0.09	0.03 ± 0.09	0.726	0.469	0.390
Plasma HDL-C (mmol/L)^1^	1.60 ± 0.11	1.72 ± 0.13	0.13 ± 0.04	1.42 ± 0.09	1.48 ± 0.10	0.09 ± 0.03	**< 0.001**	0.160	0.418
Plasma TG (mmol/L)^1^	0.90 ± 0.11	0.89 ± 0.11	− 0.01 ± 0.04	1.48 ± 0.17	1.23 ± 0.11	− 0.26 ± 0.16	0.119	**0.011**	0.161
Plasma FA (mmol/L)^1^	0.65 ± 0.06	0.54 ± 0.04	− 0.11 ± 0.06	0.57 ± 0.05	0.51 ± 0.04	− 0.06 ± 0.04	**0.029**	0.381	0.462
Plasma ApoA-I (g/L)^5^	1.56 ± 0.06	1.61 ± 0.07	0.05 ± 0.06	1.69 ± 0.07	1.59 ± 0.07	− 0.08 ± 0.05	0.667	0.649	0.104
Plasma PCSK9 (ng/mL)^1^	214 ± 23	169 ± 18	− 48.0 ± 18.2	208 ± 13	190 ± 18	− 18.5 ± 15.2	**0.009**	0.776	0.222
Plasma apoB/PCSK9 (mg/µg)^1^	4.41 ± 0.41	6.01 ± 0.67	1.60 ± 0.49	6.23 ± 0.35	6.89 ± 0.60	0.67 ± 0.59	**0.007**	**0.040**	0.236
Plasma IL-1Ra (pg/mL)^1^	389 ± 97	373 ± 40	− 28.3 ± 75.2	437 ± 49	517 ± 70	78.9 ± 46.9	0.560	0.321	0.227
Plasma-PL EPA (µmol/L)	12.7 ± 1.8	62.3 ± 10.5	49.6 ± 10.0	15.6 ± 1.5	83.0 ± 13.3	67.5 ± 13.1	**< 0.001**	0.194	0.292
Plasma-PL DHA (µmol/L)	32.0 ± 3.8	53.4 ± 8.4	21.4 ± 9.2	41.2 ± 4.8	71.9 ± 12.3	30.7 ± 12.3	**0.002**	0.113	0.554
RBC-PL EPA (µmol/L)	10.2 ± 1.0	43.2 ± 4.7	33.0 ± 4.4	12.8 ± 1.3	58.3 ± 8.5	45.5 ± 8.4	**< 0.001**	0.103	0.205
RBC-PL DHA (µmol/L)	43.3 ± 5.1	60.6 ± 7.9	17.4 ± 5.8	55.9 ± 6.3	79.4 ± 11.3	23.5 ± 9.6	**0.001**	0.121	0.595
Plasma % EPA + DHA	1.08 ± 0.09	2.90 ± 0.34	1.82 ± 0.35	1.25 ± 0.11	3.77 ± 0.55	2.53 ± 0.54	**< 0.001**	0.141	0.285
RBC % EPA + DHA	1.35 ± 0.11	2.77 ± 0.30	1.42 ± 0.24	1.87 ± 0.18	3.71 ± 0.46	1.84 ± 0.40	**< 0.001**	**0.040**	0.383
Plasma omega-6/omega-3 ratio	15.5 ± 1.9	7.7 ± 1.2	− 7.84 ± 2.32	13.7 ± 1.3	6.2 ± 1.0	− 7.46 ± 1.80	**< 0.001**	0.217	0.897
RBC omega-6/omega-3 ratio	9.15 ± 0.85	4.74 ± 0.60	− 4.41 ± 0.85	6.44 ± 0.61	3.46 ± 0.37	− 2.98 ± 0.55	**< 0.001**	**0.010**	0.164

Data are presented as average ± SEM and analyzed by 2-way ANOVA for repeated measures with interaction.

*p < 0.001 for significant difference from low-apoB group.

^1^N = 15 in the low-apoB and N = 16 in the high-apoB, ^2^N = 15 in both groups, ^3^N = 14 in the low-apoB, ^4^N = 15 in the low-apoB and N = 17 in the high-apoB, ^5^N = 14 in the low-apoB and N = 16 in the high-apoB groups for missing post-intervention data.

### Supplementation with EPA and DHA abolishes group-differences in WAT IL-1β-secretion between subjects with high- and low-apoB and inhibits LDL-induced WAT IL-1β-secretion

WAT IL-1β-secretion induced by the 7 incubation conditions with medium, native LDL, LPS and/or ATP in the 33 subjects is presented in Fig. 2. Confirming baseline data^16^, WAT IL-1β-secretion was significantly higher in subjects with high-apoB who completed the intervention despite smaller sample size (group-effect *p* = 0.014) (Fig. 2A). As hypothesized, EPA and DHA abolished group-difference in WAT IL-1β-secretion despite the lack in post-intervention changes in plasma apoB (Fig. 2B).

Moreover, at baseline, LDL alone induced IL-1β-secretion when used as a priming and activation signals of the WAT NLRP3 inflammasome in subjects with high-apoB (i.e. LDL/LDL versus medium/medium, Fig. 2C). This LDL-effect was no longer significant post-intervention (Fig. 2D). Similarly, LDL stimulated WAT IL-1β-secretion when used for priming prior to ATP in both groups (LDL/ATP vs medium/ATP) at baseline (Fig. 2E) but not post-intervention (Fig. 2F). Intriguingly, post-intervention LDL inhibited LPS-induced WAT IL-1β-secretion in all subjects (Fig. 2H).

There were no significant post-intervention changes in WAT protein expression of pro-IL-1β or WAT mRNA expression of genes related to the inflammasome subunits (*NLRP3, CASP1, IL1B*), macrophage marker (*ADGRE1*)^34^, monocyte chemoattractant protein 1 (*MCP1)*, LDL and FA receptors (*LDLR* and *CD36*), insulin sensitizing adipokine (*ADIPOQ)* or lipid metabolism and sensing (*HMGCR, SREBP1/2*) (Supplementary Fig S4). However, post-intervention WAT *MCP1* expression was lower in subjects with low-apoB. Moreover, WAT expression of *IL10,* which is upregulated upon chronic engagement of the NLRP3 inflammasome^16,35^, was higher in subjects with high-apoB. Post-intervention expression of *PPARG,* expressed in adipocytes and immune cells, was significantly decreased in both groups.

### Supplementation with EPA and DHA ameliorates T2D risk factors 

EPA and DHA increased 1st phase (Fig. 3A), 2nd phase (Fig. 3B) and total (*p* = 0.018) glucose-induced C-peptide secretion without a change in IS (Fig. 3C) or DI (Fig. 3D). Moreover, it improved postprandial plasma clearance of chylomicrons (Fig. 3E) and TG (Fig. 3F) without a group difference. Subjects with high-apoB remained with delayed postprandial plasma clearance of TG post-intervention (Fig. 3F).

As EPA and DHA are reported to have differential effects on cardiometabolic outcomes^36^, we ran a head-to-head comparison of the association of their post-intervention changes in the RBC with the changes in the measured outcomes. Post-intervention % shifts in EPA, DPA, DHA or EPA + DHA in RBC were all positively associated with % change in total C-peptide secretion_IVGTT_ (Fig. 4A–D). Conversely, the positive association with % change in IS (Fig. 4E–H) and DI (Fig. 4I–L) and negative association with that in AUC_6hrs_ plasma apoB48 (Fig. 4M–P) and TG (Fig. 4Q–T) were significant with the % shift in DHA not EPA. Importantly, while there was a positive association of % shift in DHA or EPA + DHA with the changes in plasma LDL-C (Fig. 4U–X), this appears to be driven by increased LDL-size as estimated from the LDL-C/apoB ratio (Fig. 4Y–AB). % shift in EPA + DHA was also inversely related with % change in plasma PCSK9 in the high-apoB subjects (*r* = −0.52, *p* = 0.041). % shift in EPA and/or DHA was not associated with % change in WAT IL-1β-secretion due to the large intersubject variability in the response to the intervention (Fig. 2). It was also not associated with % changes in plasma apoB, IL-1Ra, or WAT mRNA expressions of any marker (*p* > 0.05).

### Supplementation with EPA and DHA eliminates the association of LDL-induced WAT IL-1β-secretion with T2D risk factors

LDL-induced WAT IL-1β-secretion was negatively associated with 1st phase DI (Fig. 5A), total DI (*r* = − 0.64, *p* = 0.011), WAT *PPARG* mRNA (Fig. 5B) in subjects with high-apoB only who completed the trial. It was also associated negatively with WAT *CASP1* mRNA (Fig. 5C, required for adipocyte differentiation^6^) and positively with WAT *SREBP2* mRNA (Fig. 5D, upregulated upon NLRP3 inflammasome activation^4^) in all subjects. None of these associations remained significant post-intervention (Fig. 5E–H). Moreover, at baseline, LDL/ATP-induced WAT IL-1β-secretion was associated in the direction of higher diabetes risk in subjects with high-apoB, associating negatively with 1st phase and total DI (Fig. 6A and B), IS (Fig. 6C), and WAT *PPARG* mRNA (Fig. 6F) and positively with *SREBP2* mRNA (Fig. 6G) and android fat (Fig. 6I). Conversely in subjects with low-apoB, LDL/ATP-induced WAT IL-1β-secretion was associated in the direction of lower diabetes risk, associating positively with IS (Fig. 6C) and negatively with WAT expression of *ADGRE1* and *MCP1* (Fig. 6D and E). WAT IL-1β-secretion was also positively associated with BMI and total fat (Fig. 6H and J) in all subjects. These associations including adiposity-related were eliminated post-intervention (Fig. 6K–T) except for the associations with WAT expression of *ADGRE1* and *PPARG* (Fig. 6N, P).

Using microbial LPS instead of LDL for priming boosts WAT IL-1β-secretion in subjects with low-apoB to similar levels as in high-apoB revealing its association with T2D risk factors in all subjects (Fig. 7A–J). At baseline, LPS/LDL-induced WAT IL-1β-secretion was strongly correlated with increased total GIIS_IVGTT_ (Fig. 7A), reduced IS (Fig. 7B), delayed postprandial clearance of plasma chylomicrons and TG (Fig. 7C and D), reduced markers of WAT function *ADIPOQ* and *CD36* (Fig. 7E and F), increased apoB-lipoprotein numbers and reduced estimated LDL size (Fig. 7G and H). It also associated with increased total and central adiposity (Fig. 7I and J). None of these associations remained significant post-intervention (Fig. 7K–T).

Finally, baseline WAT mRNA expressions of *NLRP3* or *IL1B* were associated with systemic risk factors for T2D and markers of WAT inflammation and dysfunction without a group-difference (Supplementary Fig S5). WAT *NLRP3* expression was also positively associated with LDL-enrichment with pro-inflammatory palmitate (saturated FA) and arachidonate (omega-6 FA). Post-intervention, WAT *NLRP3* or *IL1B* expressions were not associated with IS, DI, estimated LDL size, C-peptide secretion, LDL-enrichment with palmitate or arachidonate, and/or delayed plasma clearance of TG. However, their associations with plasma HDL-C and WAT expression of *ADGRE1, SREBP2* and *PPARG* remained significant (Supplementary Fig S6).

### EPA and DHA inhibit WAT IL-1β-secretion induced by LDL, LPS and/or ATP ex vivo

We first tested the effects of increasing concentrations of EPA and DHA in unstimulated and maximally stimulated WAT in 5 subjects at baseline. All concentrations used inhibited WAT IL-1β-secretion in unstimulated WAT by about tenfold without a dose-dependent effect (Fig. 8A). Conversely, EPA and DHA inhibited LPS/ATP-induced WAT IL-1β-secretion in a dose-dependent manner reaching significance at 200 µmol/L (~100-fold inhibition, Fig. 8B). Palmitate or oleate had no effect on IL-1β-secretion (Fig. 8A and B).

We then used the concentration of 200 µmol/L to test the effects of EPA and DHA on IL-1β-secretion in 18 subjects who had sufficient WAT yield. With a larger sample size than in Fig. 8A, oleate and palmitate modestly induced IL-1β-secretion from unstimulated WAT and the inhibitory effects of EPA and DHA were confirmed (Fig. 8C). Post-intervention, only the anti-inflammatory effects of EPA and DHA remained significant (Fig. 8C). EPA and DHA also inhibited LDL-induced WAT IL-1β-secretion as priming or activation signals (Fig. 8D and E). They also inhibited maximal WAT IL-1β-secretion induced by LPS/ATP at baseline and post-intervention (Fig. 8F). Notably, a concentration of 200 µmol/L equals that of combined EPA and DHA in the blood following the intervention but represents less than 10% of oleate or palmitate concentrations (Table 1, Supplementary Fig. S2).

## Discussion

We present novel findings indicating that 12-week supplementation with EPA and DHA (1) abolishes group-differences in the activation of WAT NLRP3 inflammasome between subjects with high-apoB versus low-apoB mainly by inhibiting LDL- and ATP- induced IL-1β-secretion (Fig. 2). Intiguingly, post-intervention LDL also inhibit WAT IL-1β-secretion induced by microbial LPS (Fig. 2G and H); (2) improves GIIS and postprandial fat metabolism (Fig. 3) in correlation with the post-intervention enrichment of RBC with EPA, DPA and mostly DHA (Fig. 4); and (3) eliminates most of the associations of WAT NLRP3 inflammasome priming (mRNA expression of *NLRP3* and *IL1B*) and activation (IL-1β-secretion) with T2D and cardiometabolic risk factors including adiposity (Figs. 5, 6, and 7, Supplementary Figs. S5 and S6). Finally, ex vivo*,* EPA and DHA used at similar concentrations to those in subject blood post-intervention inhibit WAT IL-1β-secretion in a dose-dependent manner (Fig. 8).

Our work over the past 15 years has unraveled a novel role for LDL in the pathology of T2D beyond their traditional role in CVD^5^. We reported that native LDL inhibit human adipocyte differentiation and function^25^ as well as WAT function^23,28^. In line, subjects with high-apoB have WAT dysfunction and related risk factors for T2D^21–23,28,30^. Similarly, subjects with high plasma apoB/PCSK9 ratio^21^ or high WAT surface-expression of LDL receptors and CD36^25,26^ also have WAT dysfunction and related T2D risk factors. Epidemiological data confirmed that plasma apoB predicts the incidence of diabetes 3–21 years before its onset^37–40^. Moreover, human conditions with low plasma LDL-C but upregulated receptor-mediated tissue uptake of LDL have higher risk for T2D. These conditions include subjects on statin therapy^41,42^, with loss-of-function variants in *PCSK9 or HMGCR* (target of statins)^43,44^, or gain-of-function variants in *LDLR*^44^.

Baseline data of this trial suggests that this may be mediated, at least in part, by LDL-induced upregulation of the NLRP3 inflammasome/IL-1β pathway^16^. Native LDL induce *IL1B* expression and/or IL-1β-secretion in human monocyte-derived macrophages and subcutaneous WAT^16^. Accordingly, subjects with high-apoB had higher WAT IL-1β-secretion than those with low-apoB independent of multiple covariates. Statistical adjustment for IL-1β-secretion weakened the association of plasma apoB with measures of IR, hyperinsulinemia, systemic inflammation and postprandial hyperchylomicronemia^16^. Moreover, we previously reported that subjects with low plasma LDL-C and high WAT surface-expression of LDL receptor and CD36 have higher WAT IL-1β-secretion than controls with lower WAT-receptors^25^. Cumulatively, these findings suggest that targeting LDL-induced upregulation of WAT NLRP3 inflammasome/ IL-1β secretion may reduce T2D risk factors; however, this remained to be established.

Here we show that marine-source EPA and DHA treat LDL-induced upregulation of human WAT IL-1β-secretion. Published evidence points to the implication of multiple mechanisms. In vitro models including adipocytes and macrophage*,* EPA and/or DHA were shown to inhibit nuclear factor-κB and c-Jun N-terminal kinase pathways and the priming and activation of the NLRP3 inflammasome by metabolic and non-metabolic signals (palmitate, LPS, ATP)^17,18,45^. They were also reported to inhibit the expression of CD36^46^, activation of caspase-1 and cleavage of IL-1β^18^ in macrophage and to decrease macrophage-migration towards MCP-1^47^. DHA incorporation into plasma PL also decrease the dimerization and recruitment of toll-like receptor 4 induced by saturated FA or LPS into lipid raft fractions^48^. Furthermore, EPA and DHA give rise to potent inflammation-resolving bioactive molecules^33,49^. In LPS-stimulated human monocytes, these bioactive molecules inhibit the secretion of pro-inflammatory cytokines including IL-1β while increase that of anti-inflammatory IL-10^33,49^. Conversely, omega-6-derived oxylipins particularly from arachidonate, are associated with inflammation and adverse cardiometabolic outcomes^50^_._ Thus, the post-intervention decrease in pro-inflammatory omega-6 FA and omega-6/omega-3 ratio (Table 1, Fig. 1) may have further potentiated the anti-inflammatory effects of EPA and DHA.

EPA and DHA may have also modulated LDL quality. While native LDL were used in this trial without in vitro modification, the presence of oxidized LDL^51^, acetylated LDL^52^, or ceramide-enriched LDL^53^ in subject LDL preparations cannot be excluded. These components can upregulate the NLRP3 inflammasome. EPA and DHA were reported to reduce ceramide synthesis and toxicity in palmitate-loaded macrophages and animal models on high-fat diet^54^. They were also shown to reduce the uptake of acetylated LDL by human THP-1 and monocyte-derived macrophages, while DHA was also shown to reduce the uptake of oxidized LDL^46^. Furthermore, EPA and DHA are well recognized to reduce hypertriglyceridemia, which would favor reduced LDL TG-content and lipolysis and increased LDL size^50^. Intriguingly, post-intervention enrichment of RBC with DHA rather than EPA was better associated with improved T2D risk factors, including reduced IS and DI and postprandial hypertriglyceridemia (Fig. 4) despite the greater post-intervention increase in circulating EPA (Fig. 1). While the mechanisms for these findings cannot be evaluated from our trial, DHA was show to have a greater inhibitory effect than EPA on mRNA expression of *NLRP3*, *CASP1*, and *IL1B* in human subcutaneous WAT ex vivo and in THP-1 macrophages that were cocultured with human primary adipocytes^55^. Furthermore, DHA anti-inflammatory effects appear to be dependent on the NLRP3 inflammasome, as the amelioration of systemic, liver and WAT inflammation and IS by DHA is significant in wildtype but not *Nlrp3* deficient mice^18^.

**Fig. 9:**
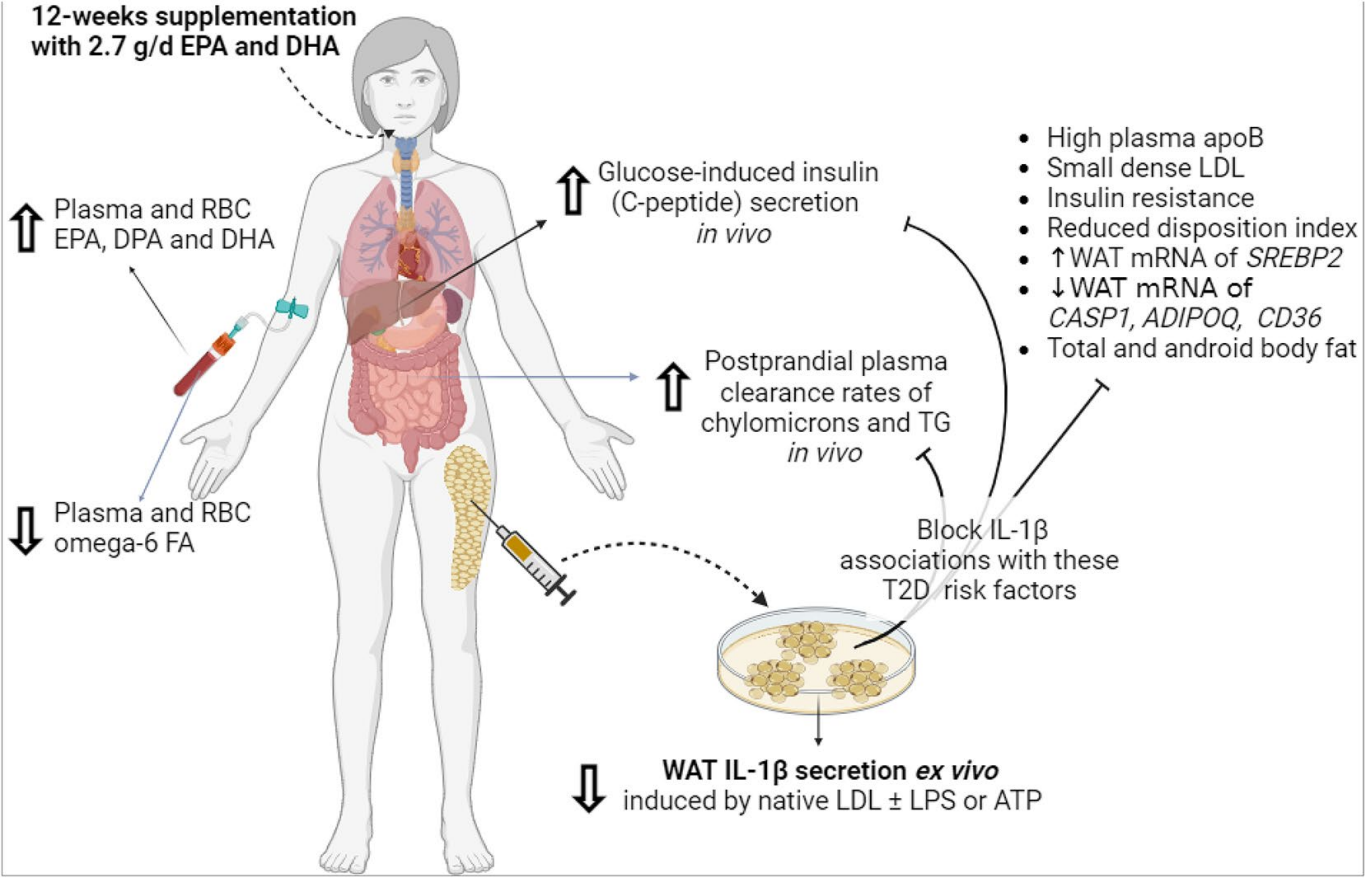
Pleiotropic effects of EPA and DHA: Supplementation with 2.7 g/d EPA and DHA over 12-weeks inhibited LDL-induced WAT-IL-1β-secretion ex vivo*,* ameliorated GIIS secretion and postprandial plasma clearance of chylomicrons and TG, and inhibited the association of WAT-IL-1β-secretion with multiple systemic risk factors for T2D and measures of WAT inflammation and dysfunction

Cumulatively, these pleiotropic effects of EPA and DHA may explain the blunted associations of the WAT NLRP3 inflammasome priming and activation with cardiometabolic risk factors (Fig. 9). Many other lines of evidence also support a role for EPA and DHA in reduced cardiometabolic disease. While elevated plasma FA was reported to be predict the incidence of T2D over 11 years, this association is significant in subjects with low (< 75th percentile) not high plasma (> 75th percentile) PL EPA and DHA^56^. A recent meta-analysis of 67 prospective studies with over 1-year follow-up reported that the risk for T2D, CVD and overall mortality was inversely associated with the enrichment of plasma, RBC and/or WAT with EPA, DPA, DHA and/or α-linolenic acid^57^. Another meta-analysis and meta-regression of 40 randomized controlled trials with EPA and/or DHA supplementation concluded a dose-dependent effect of combined EPA and DHA, ranging from 1 to 6 g/d, on CVD outcomes with no particular benefit of purified EPA^58^. In our study, no threshold was observed in the association of post-intervention RBC EPA, DPA, DHA or EPA and DHA with the amelioration in T2D risk factors (Fig. 4). Thus, the protective effects of EPA and DHA on T2D and CVD may increase with increased dosage and exposure. It remains to be tested if EPA and DHA are more effective than agents targeting IL-1β alone in the prevention of T2D in CVD patients on statin-therapy as in the CANTOS trial^59^.

Importantly, the quality of omega-3 supplementation matters. Among the most common arguments against the use of over-the-counter fish oil are their increased content of heavy metals, oxidized lipids and saturated FA and lower than labeled EPA and DHA^36,60^. These concerns were mitigated by using an Internationally Verified Omega-3-certified fish oil^20^. On the other hand, the amount of saturated FA consumed from a usual dose of fish oil ~ 1 to 5 g/day is minor compared to their total daily intake in humans particularly that only ~ 30% of fish oil is saturated^50^. In agreement, there was no post-intervention change in circulating saturated FA (Fig. 1, Supplementary Fig. S2). Moreover, we reported in baseline data of this trial that not all saturated FA have similar roles. While % plasma palmitate was associated with increased WAT *NLRP3* expression and LDL-induced IL-1β-secretion, that of stearate and myristate were associated in the reverse direction^16^.

Finally, certain strength and limitations should be outlined. The site of WAT used to assess the regulation of the NLRP3 inflammasome in highly relevant. We previously reported that higher hip WAT function is associated with higher IS (M/I_clamp_) and faster postprandial plasma clearance of chylomicrons^23^ whereas higher visceral WAT mass was associated with lower IS (M/I_clamp_)^31^. This underscores the impact of omega-3 FA or LDL on hip WAT function and related health or disease, *respectively*. On the other hand, we assessed risk factors for, and not the incidence of, T2D. Whether EPA and DHA reduce T2D incidence in subjects with high-apoB or higher receptor-mediated uptake of LDL merits further investigation.

In conclusion, supplementation with EPA and DHA eliminates group-differences in the activation of the WAT NLRP3 inflammasome as IL-1β-secretion between subjects with high-apoB and low-apoB, likely by improving the quality and pro-inflammatory profile of LDL and subcutaneous WAT. They also ameliorate glucose and fat metabolism in relation to the bioavailability of EPA, DPA and mostly DHA in the circulation (Fig. 9). Given their low-cost, availability and limited side-effects, supplementation with high-quality marine-source EPA and DHA may be an effective and sustainable lifestyle strategy for the prevention of T2D and CVD particularly among subjects with high plasma apoB.

## Supplementary Information

Supplementary Information.

## Data Availability

Data described in the manuscript will not be made publicly available due to ethical restrictions for lack of subject consent for that but are available from the corresponding author upon reasonable request.
