# Supplementary material for: EPA and DHA inhibit LDL-induced upregulation of human adipose tissue NLRP3 inflammasome/IL-1β pathway and its association with diabetes risk factors
Source: Sci Rep. 2024 Nov 7;14:27146. doi: 10.1038/s41598-024-73672-6 (PMC11543682; doi:10.1038/s41598-024-73672-6)

EPA and DHA inhibit LDL-induced upregulation of human adipose tissue NLRP3 inflammasome/IL-1β pathway and its association with diabetes risk factors

Valérie Lamantia[1,2,3], Simon Bissonnette[1,2,3], Myriam Beaudry[1,2], Yannick Cyr[1,2,3], Christine Des Rosiers[1,4], Alexis Baass[1,2,5], May Faraj[1,2,3,5]

[1] Faculty of Medicine, Université de Montréal, Montréal, Québec, [2] Institut de recherches cliniques de Montréal (IRCM), Montréal, Québec, [3] Montréal Diabetes Research Center (MDRC), Montréal, Québec, [4]Montréal Heart Institute, Montréal, Québec, [5]Faculty of Medicine, McGill University, Montréal, Québec

Supplementary Figure S1: Subject flow chart

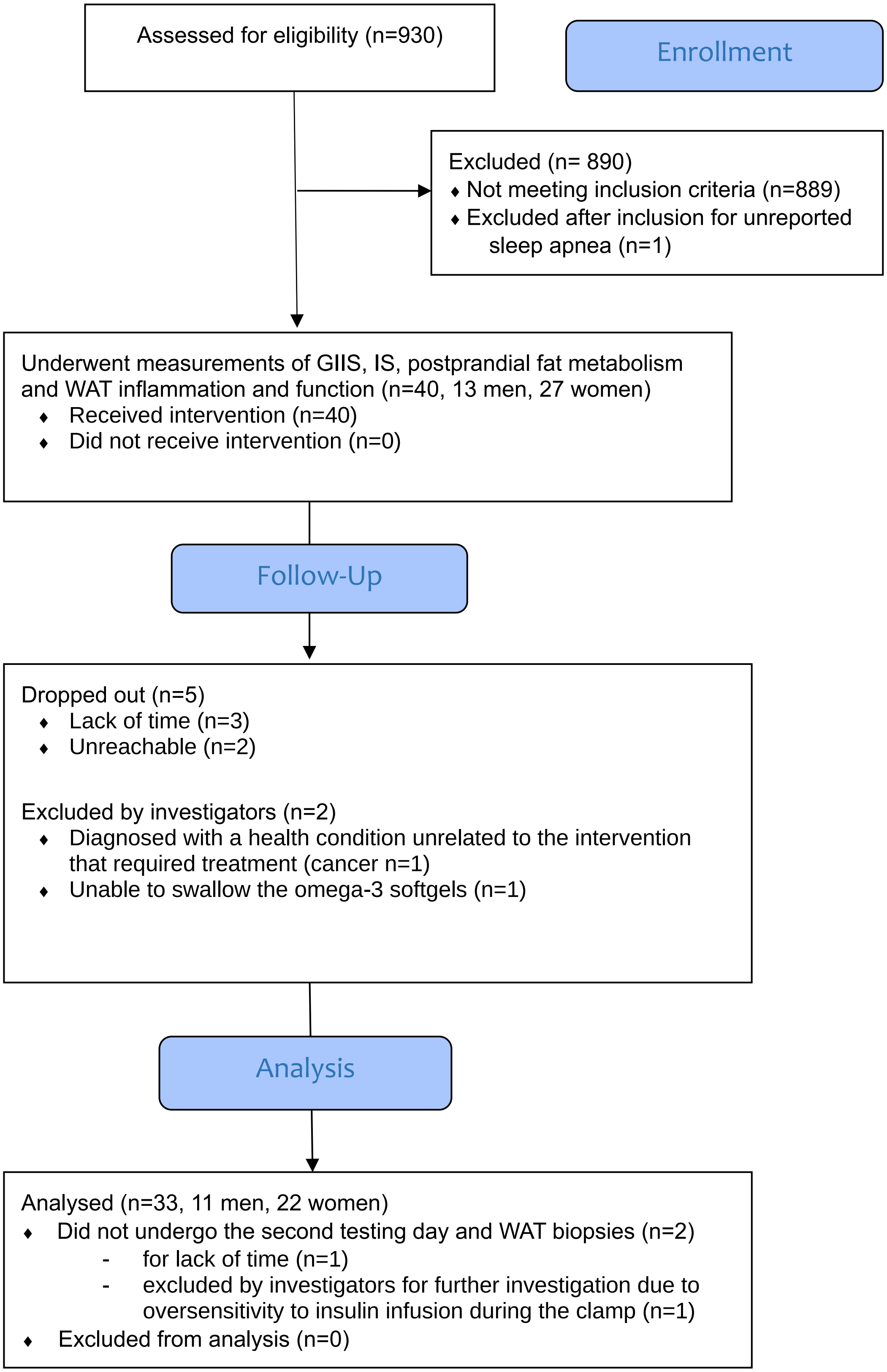

Supplementary Figure S2: Fatty acid concentrations in the phospholipid layer of A) plasma (i.e. lipoproteins) and B) red blood cells (RBC) at baseline and following the 12-week supplementation with EPA and DHA (2.7 g/d) in the 33 subjects who completed the trial. Data was analyzed as in methods. * for *p*<0.05, ** for *p*<0.01 and *** for *p*<0.001 versus baseline. N.B. Mead acid was excluded from analysis as only N=2 subjects had measurable post-intervention concentration in RBC-PL.

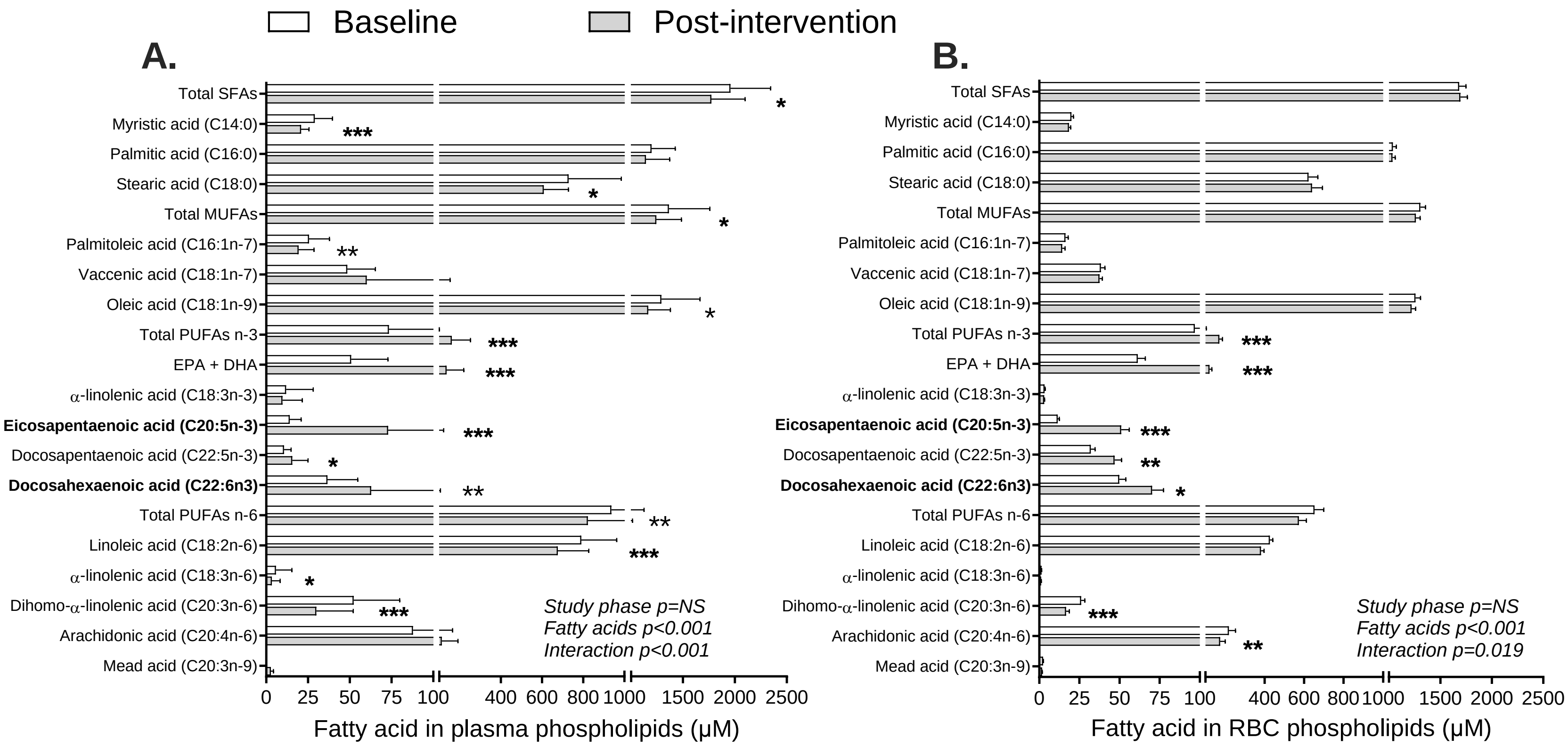

Supplementary Figure S3: Pearson correlation between post-intervention RBC and plasma phospholipid % EPA (**A**), % DHA (**B**), % EPA and DHA (**C**), % total omega-3 (**D**), % total omega-6 (**E**), and omega-6/omega-3 ratio (**F**) in subjects with low-apoB (N=16, open circles, dotted regression line) and high-apoB (N=17, closed circles, dashed regression line) who completed 12-week supplementation with EPA and DHA. Solid regression line represents pooled data for all subjects.

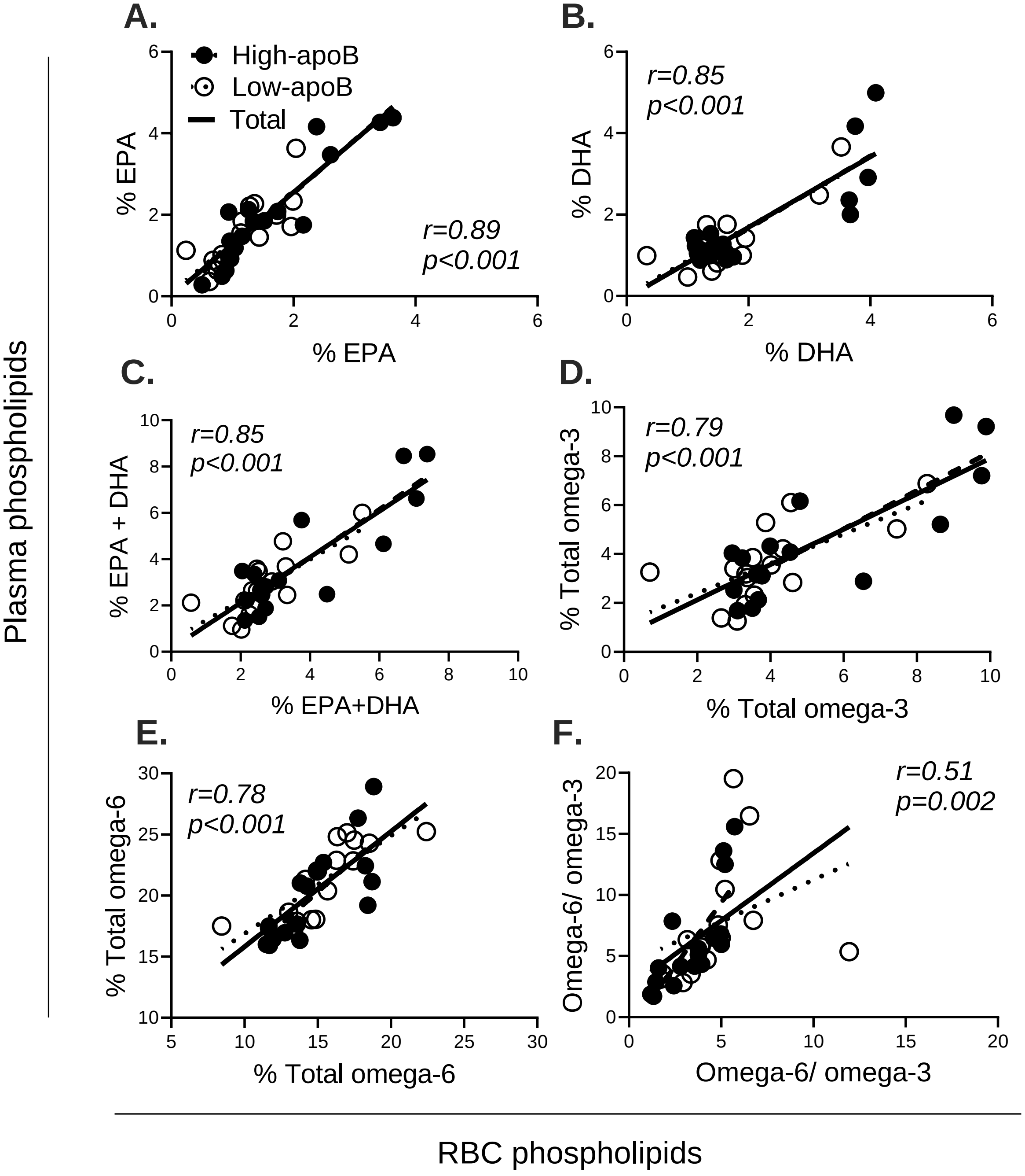

Supplementary Figure S4: Baseline and post-intervention WAT protein expression of pro-IL-1β (**A**) and WAT mRNA expression of *IL1B* (**B**), *NLRP3* (**C**), *CASP1* (**D**). *ADGRE1* **(E)**, *MCP1* (**F**), *IL10* (**G**), *LDLR* (**H**), *CD36* (**I**), *ADIPOQ* (**J**), *PPARG* (**K**), *HMGCR* (**L**) *SREBP1c* (**M**) and *SREBP2* (**N**) normalized to *HPRT* in subjects with low-apoB (baseline N=16, post-intervention N=14) and high-apoB (baseline N=17, post-intervention N=16) for missing data. Data was analyzed as in methods.

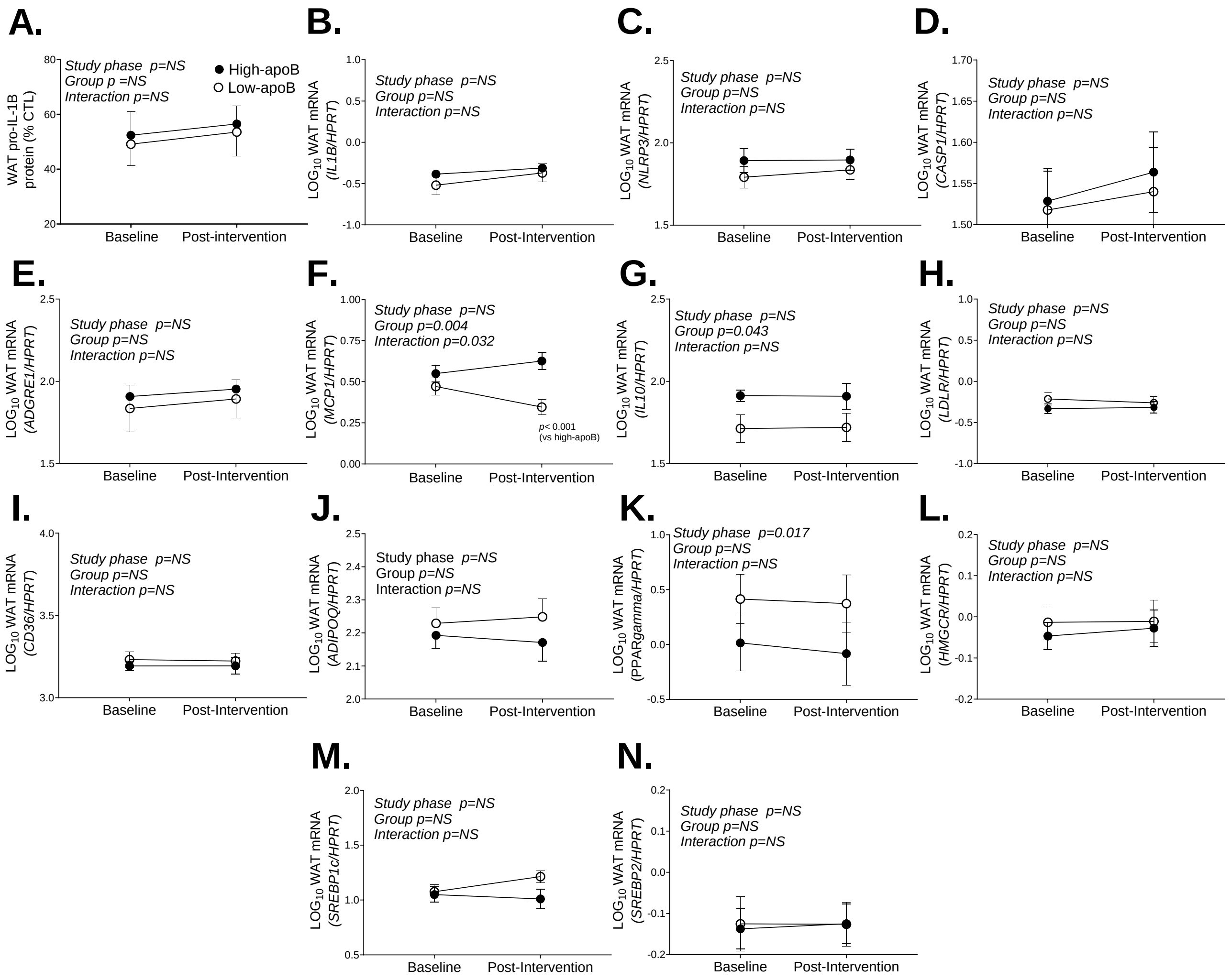

Supplementary Figure S5: Pearson correlation of fasting baseline WAT mRNA expression of *NLRP3* normalized for *HPRT* with insulin sensitivity as M/I$_{clamp}$ (**A**), total disposition index (total C-peptide$_{IVGTT}$ x M/I$_{clamp}$) (**B**), AUC$_{6hrs}$ plasma TG (**C**), fasting plasma TG (**D**), estimated fasting LDL size (**E**), fasting plasma HDL-C (**F**), and fasting WAT mRNA expression of *IL1B* (**G**), *ADGRE1* (**H**), *MCP1* (**I**), *CD36* (**J**), *ADIPOQ* (**K**), *PPARG* (**L**), and *SREBP2* (**M**) normalized to *HPRT*, and % fasting plasma phospholipid palmitate (**N**), and arachidonate (**O**). Also presented is Pearson correlation of fasting baseline WAT mRNA expression of *IL1B* normalized for *HPRT* with 1st phase (**P**), 2nd phase (**Q**), and total C-peptide secretion$_{IVGTT}$ (**R**), and fasting baseline WAT mRNA expression of *ADGRE1* normalized to *HPRT* (**S**) in subjects with low-apoB (N=16, open circles, dotted regression line) and high-apoB (N=17, closed circles, dashed regression line) who completed the trial. Solid regression line represents pooled data for all subjects.

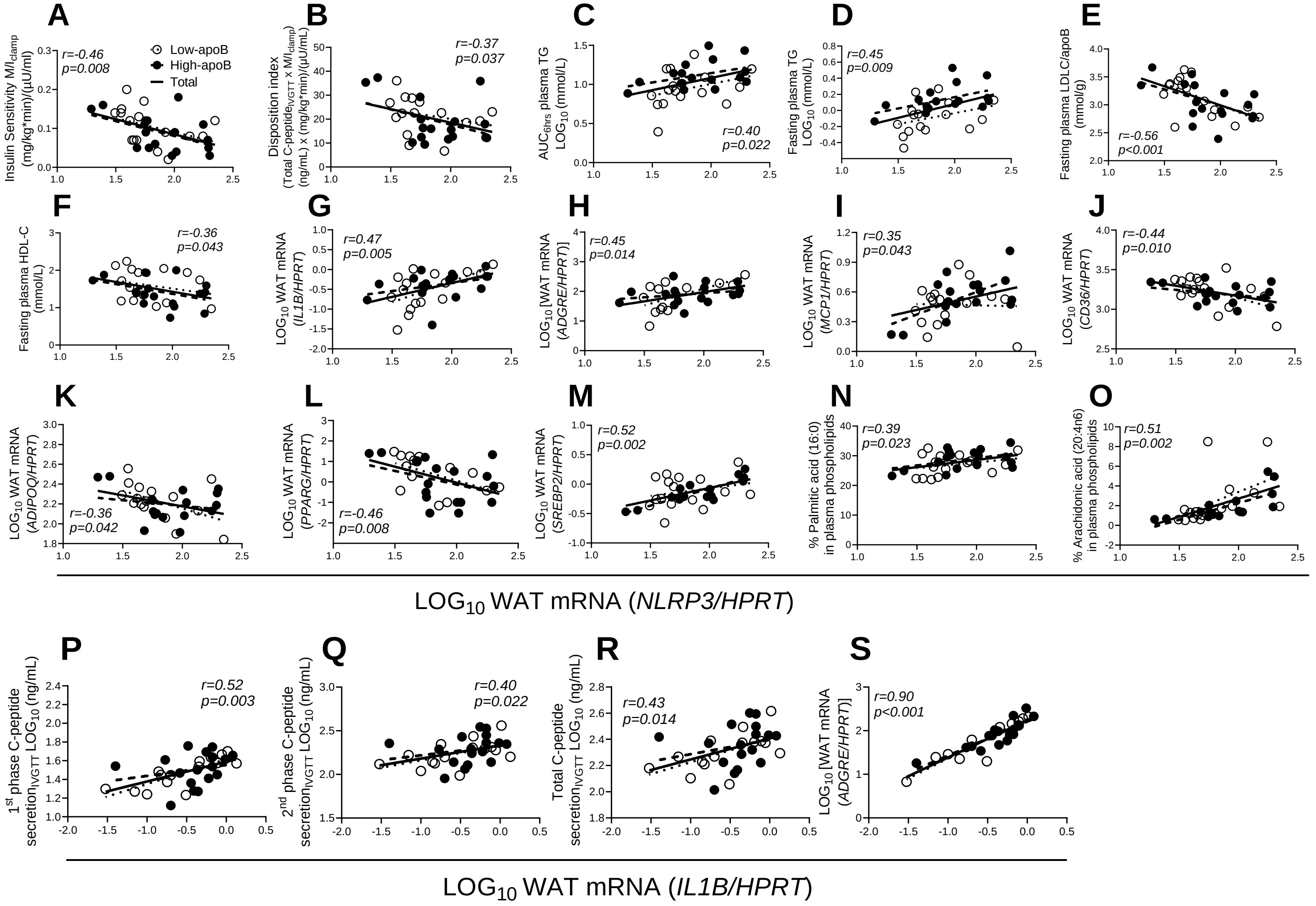

Supplementary Figure S6: Pearson correlation of fasting post-intervention WAT mRNA expression of *NLRP3* normalized for *HPRT* with insulin sensitivity as M/I_{clamp} (**A**), total disposition index (total C-peptide$_{IVGTT}$ x M/I_{clamp}) (**B**), AUC_{6hrs} plasma TG (**C**), fasting plasma TG (**D**), estimated fasting LDL size (**E**), fasting plasma HDL-C (**F**), and WAT mRNA expression of *IL1B* (**G**), *ADGRE1* (**H**), *MCP1* (**I**), *CD36* (**J**), *ADIPOQ* (**K**), *PPARG* (**L**), and *SREBP2* (**M**) normalized to *HPRT*, and % fasting plasma phospholipid palmitate (**N**), and arachidonate (**O**). Also presented is Pearson correlation of fasting post-intervention WAT mRNA expression of *IL1B* normalized for *HPRT* with 1st phase (**P**), 2nd phase (**Q**), total C-peptide secretion$_{IVGTT}$ (**R**), and post-intervention WAT mRNA expression of *ADGRE1* normalized to *HPRT* (**S**) in subjects with low-apoB (N=15, open circles, dotted regression line) and high-apoB (N=1, closed circles, dashed regression line) who completed the trial and have post-intervention WAT data. Solid regression line represents pooled data for all subjects.

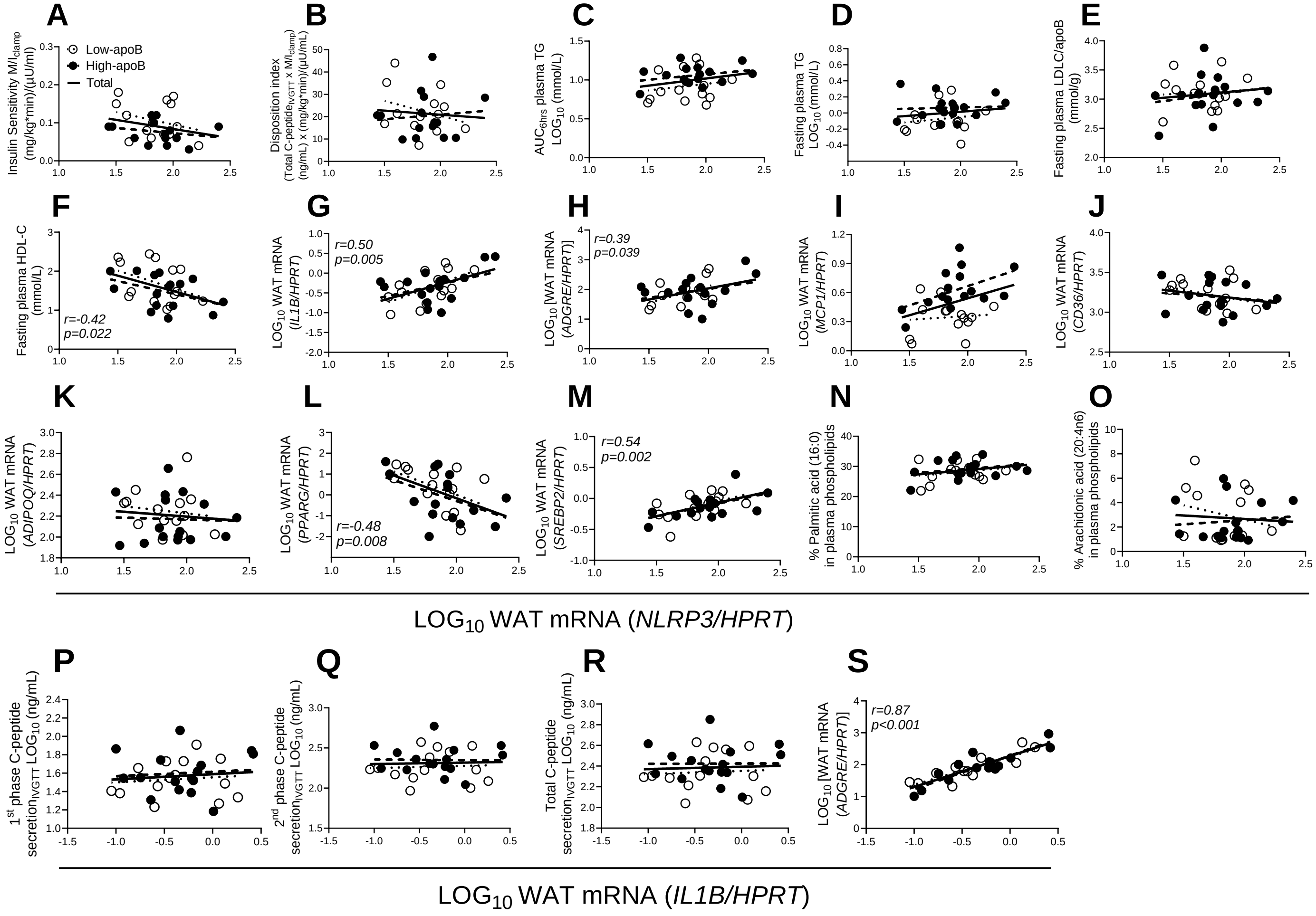

Supplement: Supplementary file 1 — Supplementary Information. [file 41598_2024_73672_MOESM1_ESM.pdf]
